# EGFRvIII-Specific Chimeric Antigen Receptor T Cells Migrate to and Kill Tumor Deposits Infiltrating the Brain Parenchyma in an Invasive Xenograft Model of Glioblastoma

**DOI:** 10.1371/journal.pone.0094281

**Published:** 2014-04-10

**Authors:** Hongsheng Miao, Bryan D. Choi, Carter M. Suryadevara, Luis Sanchez-Perez, Shicheng Yang, Gabriel De Leon, Elias J. Sayour, Roger McLendon, James E. Herndon, Patrick Healy, Gary E. Archer, Darell D. Bigner, Laura A. Johnson, John H. Sampson

**Affiliations:** 1 Duke Brain Tumor Immunotherapy Program, Division of Neurosurgery, Department of Surgery, Duke University Medical Center, Durham, North Carolina, United States of America; 2 Department of Pathology, Duke University Medical Center, Durham, North Carolina, United States of America; 3 Department of Molecular Cancer Biology, Duke University Medical Center, Durham, North Carolina, United States of America; 4 The Preston Robert Tisch Brain Tumor Center, Duke University Medical Center, Durham, North Carolina, United States of America; 5 Department of Biostatistics and Bioinformatics, Duke University Medical Center, Durham, North Carolina, United States of America; University of Michigan School of Medicine, United States of America

## Abstract

Glioblastoma (GBM) is the most common primary malignant brain tumor in adults and is uniformly lethal. T-cell-based immunotherapy offers a promising platform for treatment given its potential to specifically target tumor tissue while sparing the normal brain. However, the diffuse and infiltrative nature of these tumors in the brain parenchyma may pose an exceptional hurdle to successful immunotherapy in patients. Areas of invasive tumor are thought to reside behind an intact blood brain barrier, isolating them from effective immunosurveillance and thereby predisposing the development of "immunologically silent" tumor peninsulas. Therefore, it remains unclear if adoptively transferred T cells can migrate to and mediate regression in areas of invasive GBM. One barrier has been the lack of a preclinical mouse model that accurately recapitulates the growth patterns of human GBM *in vivo.* Here, we demonstrate that D-270 MG xenografts exhibit the classical features of GBM and produce the diffuse and invasive tumors seen in patients. Using this model, we designed experiments to assess whether T cells expressing third-generation chimeric antigen receptors (CARs) targeting the tumor-specific mutation of the epidermal growth factor receptor, EGFRvIII, would localize to and treat invasive intracerebral GBM. EGFRvIII-targeted CAR (EGFRvIII^+^ CAR) T cells demonstrated *in vitro* EGFRvIII antigen-specific recognition and reactivity to the D-270 MG cell line, which naturally expresses EGFRvIII. Moreover, when administered systemically, EGFRvIII^+^ CAR T cells localized to areas of invasive tumor, suppressed tumor growth, and enhanced survival of mice with established intracranial D-270 MG tumors. Together, these data demonstrate that systemically administered T cells are capable of migrating to the invasive edges of GBM to mediate antitumor efficacy and tumor regression.

## Introduction

Glioblastoma (GBM) is the most common form of primary malignant brain tumor in adults and remains one of the most deadly neoplasms. Despite multimodal therapy including maximal surgical resection, radiation, and temozolomide (TMZ), the median overall survival is less than 15 months [1]. Moreover, these therapies are non-specific and are ultimately limited by toxicity to normal tissues [2]. In contrast, immunotherapy promises an exquisitely precise approach, and substantial evidence suggests that T cells can eradicate large, well-established tumors in mice and humans [3]–[7].

Chimeric antigen receptors (CARs) represent an emerging technology that combines the variable region of an antibody with T-cell signaling moieties, and can be genetically expressed in T cells to mediate potent, antigen-specific activation. CAR T cells carry the potential to eradicate neoplasms by recognizing tumor cells regardless of major histocompatibility complex (MHC) presentation of target antigen or MHC downregulation in tumors, factors which allow tumor-escape from treatment with *ex vivo* expanded tumor-infiltrating lymphocytes (TILs) [8] and T-cell receptor (TCR) gene therapy [9], [10]. Clinical trials utilizing CARs in other tumor systems including renal cell carcinoma [11], indolent B-cell and mantle cell lymphoma [12], neuroblastoma [13], acute lymphoblastic leukemia [14], and chronic lymphoid leukemia [15] have verified their remarkable potential. However, severe adverse events, including patient deaths, have occurred from administration of CAR T cells when directed against tumor antigens simultaneously expressed on normal tissues [16], [17].

The tumor-specific variant of the epidermal growth factor receptor, EGFRvIII, is a type III in-frame deletion mutant of the wild-type receptor that is exclusively expressed on the cell surface of GBMs and other neoplasms but is absent on normal tissues [18]–[20]. Unlike previous CARs, an EGFRvIII-specific construct carries the potential to eliminate tumor cells without damaging normal tissue due to the tumor specificity of its target antigen. Thus, as a tumor-specific CAR, EGFRvIII-targeted CARs (EGFRvIII^+^ CARs) should be able to employ the previously demonstrated potency of CAR T cells both precisely and safely against tumor when implemented into the clinic.

Despite their promise, the utility of CAR therapy against brain tumors has been questioned due to the concept of central nervous system (CNS) immune privilege. This dogma has since been challenged, as T cells are now known to infiltrate CNS parenchyma in the context of neuropathology and neuroinflammation where the blood brain barrier (BBB) is known to be disrupted [21], [22]. GBM in particular has been implicated in BBB dysfunction through its modulation of the local brain microenvironment, owing in part to both the inevitable disruption of natural brain architecture by bulky tumor masses and their inherent pathologic characteristics that increase the permeability of microvessels, thereby compromising BBB integrity [23]. While it is reasonable to suspect that T cells and chemotherapeutic agents may gain entry to tumor cores through these regions of increased permeability, the long-term therapeutic benefits of this rationale have been marred by the fact that GBM is predisposed to the development of highly invasive neoplastic peninsulas that are removed from main tumor masses, residing within normal brain areas that are protected by regions of intact BBB [24]–[26]. This may explain the failure of therapeutic regimens that depend on BBB permeability for targeted treatment delivery, where main tumor cores are discriminately subjected to therapy while invasive tumor cells are able to evade clinical intervention and tumor recurrence becomes inevitable [27].

It recent years, preclinical evaluations of GBM therapy have correlated only poorly with their clinical counterpart [28], and it has been increasingly difficult to reconcile this apparent discrepancy in efficacy. One explanation may be the lack of a suitable preclinical GBM model in which tumor engraftment adequately mimics the invasive features and physiological growth patterns found in the clinical scenario. Human tumor xenografts are often criticized due to their production of large, well-circumscribed, non-invasive intracranial masses (e.g. U87MG [29]–[31]), characteristics that impede their use as an adequate platform for evaluating novel therapies against GBM. Thus, identifying a preclinical model that accurately recapitulates human GBM is a critical first step to evaluating the efficacy of novel therapies designed to target highly invasive gliomas arising *de novo* in the brain.

To address these issues in the current study, we developed a model system to examine the efficacy of CAR T-cell therapy using a brain tumor explant that more precisely reflects clinically relevant growth patterns of GBM. D-270 MG is a tumor line derived directly from a patient’s primary GBM by direct orthotopic transplant and is known to naturally express EGFRvIII [32]. In order to monitor the *in vivo* efficacy of EGFRvIII^+^ CAR T cells against D-270 MG intracranial xenografts, we produced a cell line that co-expresses firefly luciferase (FLuc) and GFP, D-270MG^FLuc/GFP^, which retains the pathological features of human GBM and displays homogeneous levels of EGFRvIII expression. Here, we present data to demonstrate that D-270MG^FLuc/GFP^ xenografts display the invasive nature and hallmark characteristics of human GBM, making it an ideal preclinical model for the evaluation of this CAR-based strategy. We show that EGFRvIII^+^ CAR T cells are capable of recognizing D-270MG^FLuc/GFP^ cells in an antigen-specific manner *in vitro* and are capable of migrating into the invasive edges of intracerebral D-270MG^FLuc/GFP^ tumors in NOD.Cg-*Prkdc^scid^ Il2rg^tm1Wjl^*/SzJ (NSG) mice. Importantly, our results indicate that treatment of D-270MG^FLuc/GFP^ tumors with EGFRvIII^+^ CAR T cells significantly inhibited tumor growth and prolonged survival in NSG mice. Taken together, these observations in our novel model system demonstrate that adoptively transferred EGFRvIII^+^ CAR T cells can readily traffic *in vivo* to the invasive edges of GBM to mediate antigen-specific tumor regression.

## Results

### D-270MG^FLuc/GFP^ xenograft is highly invasive in NSG mice

**Figure 1:**
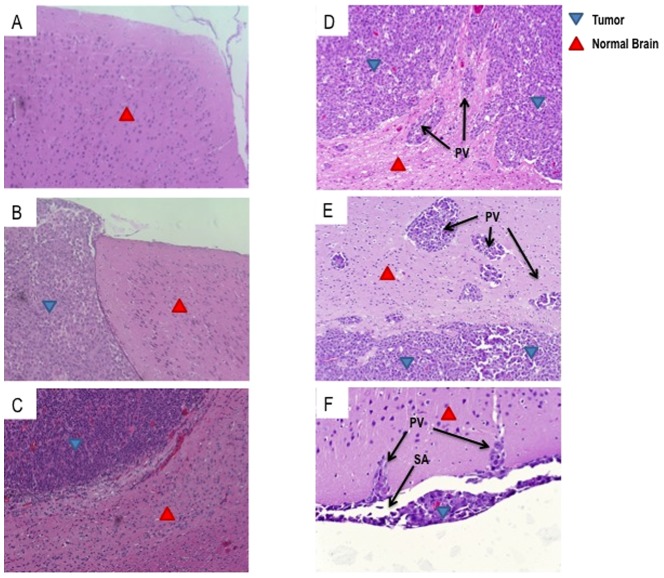
D-270MG^FLuc/GFP^ xenograft is highly invasive in NSG mice. NSG mice received intracerebral tumor implants (D-270MG^FLuc/GFP^/1×10^4^ cells or U87MG.ΔEGFR/1×10^4^ cells) and were sacrificed after seven days. Histological analysis by H&E staining of (**a**) non-tumor bearing brain, (**b, c**) established U87MG.ΔEGFR and (**d, e, f**) D-270MG^FLuc/GFP^ intracerebral malignant gliomas in NSG mice is shown. Figures delineate tumor vs. normal brain and demonstrate perivascular (PV) and subarachnoid (SA) infiltration. Images are representative of tumors obtained and analyzed from six mice (n = 6). x20 magnification.

In order to evaluate the antitumor efficacy of systemically delivered EGFRvIII^+^ CAR T cells against invasive intracerebral GBM tumors, we utilized the D-270MG^FLuc/GFP^ xenograft, which was isolated directly from a patient’s primary GBM tumor and has been previously validated to naturally express EGFRvIII [20], [32]. We first sought to histologically evaluate and compare the characteristic growth patterns of D-270MG^FLuc/GFP^ tumor with U87MG.ΔEGFR, a xenograft derived from a previously described human glioma cell subline [33], [34] that is among the most frequently used models in preclinical studies of GBM. U87MG.ΔEGFR is an EGFRvIII^+^ stably transfected subline of the parental human malignant astrocytoma cell line U87MG, which does not naturally express EGFRvIII. NSG mice received intracerebral tumor implants and were sacrificed after seven days. Brains were formalin-fixed, paraffin-embedded, and 5 μm sections were stained with hematoxylin and eosin (H&E). NSG mice with no tumors were used as a control (**Fig. 1a**). Here, we show that U87MG.ΔEGFR xenografts produce tumors with well-defined boundaries that can be clearly delineated from normal brain (**Fig. 1b, c**). In stark contrast, D-270MG^FLuc/GFP^ tumors exhibit expanding borders with small perivascular streams of cells radiating from central tumor masses, and even subarachnoid infiltration of tumor cells (**Fig. 1d, e, f**). We found that D-270MG^FLuc/GFP^ xenografts reveal an invasive nodular proliferation of malignant cells with reduced eosinophilic cytoplasm and large nuclei with prominent nucleoli. We also observed focal areas of necrosis and frequent mitotic activity in tumors. Altogether, these data demonstrate that the *in vivo* phenotype of D-270MG^FLuc/GFP^ xenografts is consistent with the classic features of human GBM. Moreover, the *in vivo* growth patterns of this model recapitulate the diffuse and infiltrative nature of tumors found in patients, making D-270MG^FLuc/GFP^ an exemplary model for evaluating this CAR-based platform.

### T cells expressing EGFRvIII^+^ CARs recognize D-270MG^FLuc/GFP^ tumors that naturally express EGFRvIII

**Figure 2:**
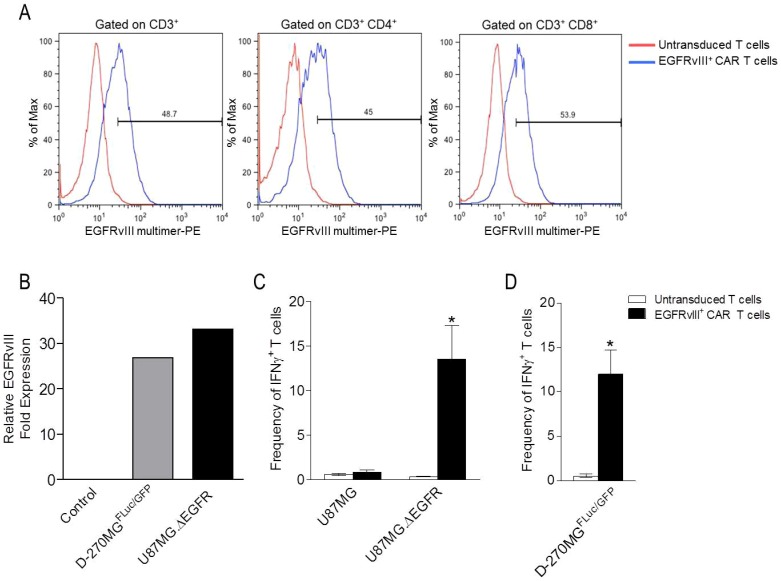
T cells expressing EGFRvIII^+^ CARs recognize D-270MG^FLuc/GFP^ tumors that naturally express EGFRvIII. (**a**) Cells were stained for EGFRvIII^+^ CARs to detect cell-surface expression using EGFRvIII multimer-PE. Negative staining controls were conducted by staining untransduced T cells from the same donor. EGFRvIII CAR^+^ T cells were gated on CD3^+^ (left), CD3^+^ CD4^+^ (middle), and CD3^+^ CD8^+^ (right). (**b**) Expression levels of EGFRvIII were quantified in D-270MG^FLuc/GFP^ and U87MG.ΔEGFR tumor cells using qRT PCR. U87MG (EGFRvIII^-^) tumor cells were used as a control. (**c**) In order to assess EGFRvIII specificity, untransduced T cells or EGFRvIII^+^ CAR T cells were co-cultured with U87MG or U87MG.ΔEGFR tumor cells. Quantification of cells positive for ICS of IFN-γ^+^ is shown. The effect of T-cell transduction on frequency of IFN-γ^+^ cells significantly differs between U87MG and U87MG.ΔEGFR tumor cells (P = 0.0004; two-way ANOVA). (**c**) To evaluate D-270MG^FLuc/GFP^ tumor cell recognition, untransduced T cells or EGFRvIII^+^ CAR T cells were co-cultured with D-270MG^FLuc/GFP^. Quantification of cells positive for ICS of IFN-γ^+^ is shown. The effect of T-cell transduction on frequency of IFN-γ^+^ cells significantly differs between untransduced T cells and EGFRvIII^+^ CAR T cells (P = 0.002; one-way ANOVA). EGFRvIII^+^ CAR T cells were also cultured alone (no target) as a control, and quantification of cells positive for ICS of IFN-γ^+^ was negligible (data not shown). Data represent one of two (n = 2) experiments with similar results.

Effective T-cell recognition and antitumor activity requires antigen-specific receptor expression and engagement of tumorigenic antigens. Therefore, we sought to determine if EGFRvIII^+^ CAR T cells would recognize D-270MG^FLuc/GFP^ tumor cells, given their natural expression of EGFRvIII. Towards this end, we obtained human peripheral blood lymphocytes (PBLs) from patients and transduced them with a previously-described retrovirus encoding a third-generation EGFRvIII^+^ CAR containing the humanized 139 anti-human EGFRvIII single-chain variable fragment in tandem with the hCD28-41BB-CD3æ chain signaling domain [35]. Following transduction, we determined surface expression by flow cytometry, and T cells were found to efficiently express the EGFRvIII^+^ CAR construct on their cell surface (**Fig. 2a**). In order to assess antigen-specificity of EGFRvIII^+^ CAR T cells, we first quantitatively assessed levels of EGFRvIII expression in D-270MG^FLuc/GFP^, U87MG.ΔEGFR (EGFRvIII^+^), and U87MG (EGFRvIII^-^) control tumor cells. We found similar levels of EGFRvIII expression between D-270MG^FLuc/GFP^ and U87MG.ΔEGFR tumor cells (**Fig. 2b**). Next, antigen-specific reactivity against EGFRvIII was determined in co-culture assays; while EGFRvIII^+^ CAR T cells did not secrete observable levels of the type 1 cytokine interferon-gamma (IFN-γ) in response to the U87MG (EGFRvIII^-^) tumor cell line, they did produce IFN-γ in the presence of the U87MG.ΔEGFR (EGFRvIII^+^) cell line as measured by intracellular staining (ICS) (**Fig. 2c** **;** P = 0.0004; two-way ANOVA). Untransduced T cells alone showed no IFN-γ production versus either target. Importantly, recognition of the D-270MG^FLuc/GFP^ cell line was observed only by EGFRvIII^+^ CAR T cells and not by untransduced T cells (**Fig. 2d**; P = 0.002; one-way ANOVA). These data corroborate the specificity of EGFRvIII^+^ CAR T cells and demonstrate their ability to elicit antitumor activity upon interaction with D-270MG^FLuc/GFP^ tumors.

### EGFRvIII^+^ CAR T cells effectively migrate to invasive GBM tumors

**Figure 3:**
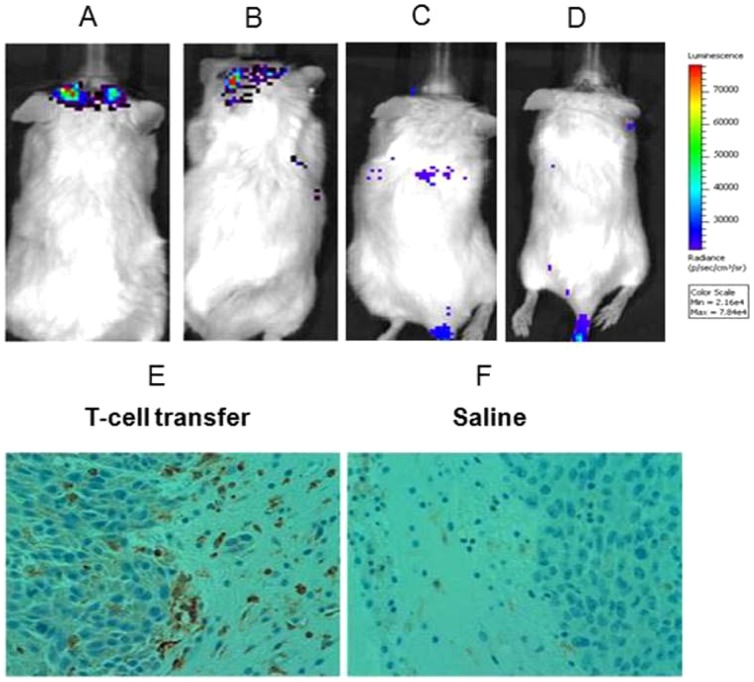
EGFRvIII^+^ CAR T cells effectively migrate to invasive GBM tumors. 1×10^7^ extGLuc^+^ EGFRvIII^+^ CAR T cells were administered systemically to D-270MG^FLuc/GFP^ tumor-bearing mice, and T-cell trafficking and/or accumulation near tumor was monitored using BLI on days 2 (**a**) and 9 (**b**). To assess the role of antigen-specificity on T-cell localization at the site of tumor, 1×10^7^ extGLuc-only T cells were systemically administered to a separate group of tumor-bearing mice and monitored for trafficking and/or accumulation near tumor using BLI on day 7 (**c, d**). NSG mice treated with extGLuc^+^ EGFRvIII^+^ CAR T cells (**e**) or saline (**f**) were sacrificed on day 9, and brains were harvested, formalin-fixed, and paraffin-embedded. 5 μm coronal sections were immunostained with rabbit anti-human CD3 antibody and counterstained with hematoxylin. Images are representative of tumors obtained and analyzed from four mice (n = 4). Data represent one of two (n = 2) experiments with similar results.

Effective therapy in a clinical setting requires that systemically administered T cells effectively migrate to and encounter tumor cells *in vivo*. Unlike cancers of the periphery, GBM tumors rarely metastasize outside of the brain, instead shedding neoplastic cells that migrate away from main tumor cores and develop into highly invasive peninsulas residing within the normal brain, hiding within regions of intact BBB [24]–[26]. Although T cells are able to access bulky GBM lesions through dysfunction of the local BBB, it is unknown if they can effectively migrate into the invading tumor deposits that may reside behind an intact BBB. Therefore, we sought to evaluate the capacity of EGFRvIII^+^ CAR T cells to localize to D-270MG^FLuc/GFP^ intracerebral tumors, which closely mirror the invasive architecture of human GBM (**Fig. 1d–f**). To examine this, human donor PBLs were transduced with the external *Gaussian* luciferase (extGLuc) retrovirus or underwent a dual transduction with both the extGLuc and EGFRvIII^+^ CAR retroviruses for use in bioluminescence imaging (BLI) analysis [36]. Following transduction, T cells were cultured *in vitro* prior to systemic infusion into NSG mice bearing established orthotopic D-270MG^FLuc/GFP^ tumors. Imaging analysis two (**Fig. 3a**) and nine (**Fig. 3b**) days after T-cell injection revealed extGLuc signals in the brain area, demonstrating localization of EGFRvIII^+^ CAR T cells to the tumor site. We also sought to evaluate whether EGFRvIII-specificity was necessary for efficient T-cell localization to tumor, and to do this, we compared the trafficking patterns of extGluc^+^ EGFRvIII^+^ T cells (**Fig. 3a,b**) with extGluc^+^ T cells (**Fig. 3c,d**) in NSG mice bearing D-270MG^FLuc/GFP^ tumors. We found that extGluc-only T cells (EGFRvIII^-^) failed to efficiently migrate across the BBB, whereas extGluc^+^ EGFRvIII^+^ CAR T cells rapidly localized in the brain. This suggests a necessity of EGFRvIII^+^ CAR expression for brain-trafficking or accumulation in NSG mice bearing D-270MG^FLuc/GFP^ tumors. A separate group of mice were also sacrificed nine days after T-cell injection and tumor tissue was submitted for immunohistochemical analysis. **Figure 3E** shows that systemically administered EGFRvIII^+^ CAR T cells successfully migrated to the invasive edges of intracerebral tumor, particularly in areas of peninsula formation at the leading edge of tumor invasion. NSG mice receiving saline were used as a control (**Fig. 3F**). These results demonstrate that, in our model of invasive GBM, adoptively transferred EGFRvIII^+^ CAR T cells have the capacity to traffic to invasive areas of tumor thought to reside beyond the BBB.

### *In vivo* systemic delivery of EGFRvIII^+^ CAR T cells delays tumor growth and prolongs survival

**Figure 4:**
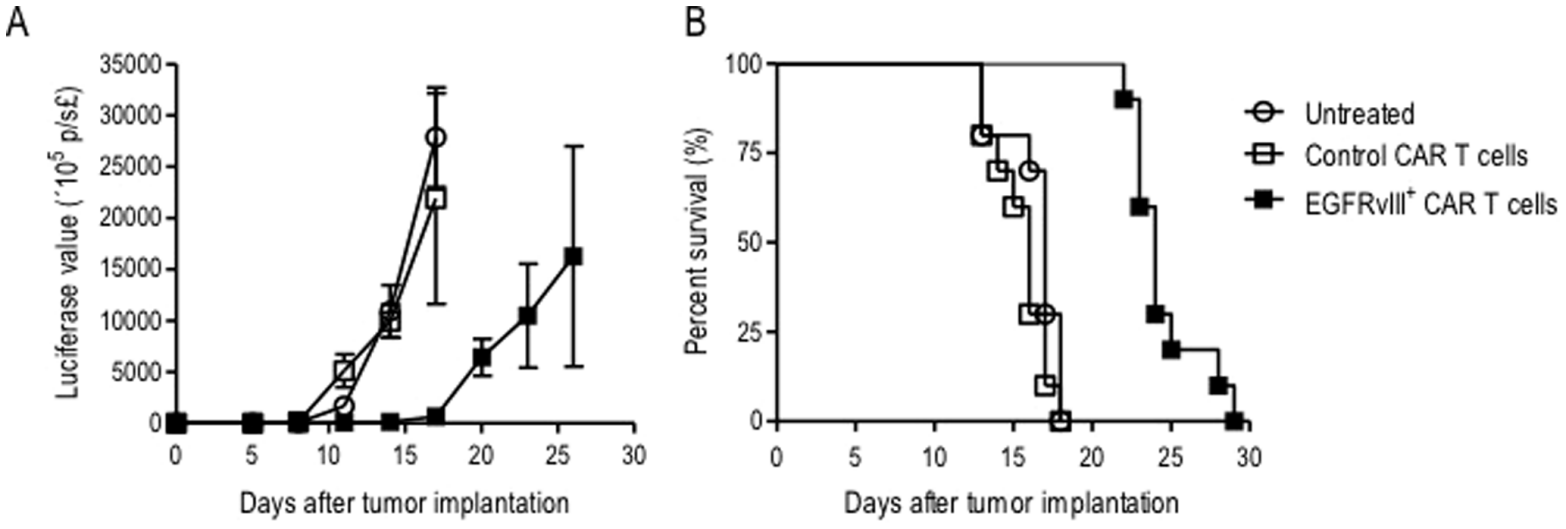
*In vivo* systemic delivery of EGFRvIII^+^ CAR T cells delays tumor growth and prolongs survival. NSG mice were implanted with 1×10^4^ D-270MG^FLuc/GFP^ tumor cells intracranially, randomized into three groups (n = 6-8), and monitored for tumor growth and survival. 5.0 - 10×10^6^ T cells were administered intravenously 3 days after tumor implantation. (**a**) Normalized BLI values associated with longitudinal monitoring of tumor growth for untreated, control CAR T-cell, and EGFRvIII^+^ CAR T-cell groups are shown (value = mean ±SD). The pattern of change in log BLI values significantly differs between the three treatment groups (P<0.0001; mixed model). (**b**) The survival of animals treated with EGFRvIII^+^ CAR T cells was significantly prolonged (P<0.0001; generalized Wilcoxon test) when compared to other treatment groups. Data represent one of two (n = 2) experiments with similar results.

We sought to determine the therapeutic effect of systemically administered EGFRvIII^+^ CAR T cells against invasive intracerebral gliomas *in vivo*. Utilizing the NSG mouse model, D-270MG^FLuc/GFP^ xenografts were implanted intracerebrally and allowed to engraft for three days prior to intravenous infusion with EGFRvIII^+^ CAR T cells. D-270MG^FLuc/GFP^ cells were monitored using BLI every three days. No significant difference was observed between groups of mice that were either left untreated or infused with non-specific control CAR T cells. However, there was a significant delay in tumor growth in EGFRvIII^+^ CAR T-cell treatment groups compared to untreated and control CAR T cells, as detected by serial BLI recordings of tumor-cell photon emissions (**Fig. 4a**; P<0.0001; mixed model). Tumors were not visible in any group until day 11, at which point tumors began growing in control and untreated mice. Complete suppression of tumor growth was evident in mice treated with EGFRvIII^+^ CAR T cells until day 17, but grew to achieve BLI values similar to untreated mice by day 26 (**Fig. 4a**). These growth kinetics translated into an 8–9 day survival advantage in mice treated with EGFRvIII^+^ CAR T cells when compared to both untreated mice and those receiving control CAR T cells (**Fig. 4b**; P<0.0001; generalized Wilcoxon test). Together, these preclinical results suggest that systemically administered third-generation EGFRvIII^+^ CAR T cells can inhibit invasive brain tumor growth and prolong survival.

## Discussion

The therapeutic benefits of CAR-based adoptive cell therapy have been widely demonstrated in patients suffering with cancer [7], [13]–[15]. Since its introduction, CAR design has evolved significantly to mediate a potent and robust T-cell immune response when directed against tumors in the periphery [6], [37]. Importantly, we demonstrate here that similar immune responses can be achieved against established tumors in the immunologically privileged brain, even when directed against highly invasive cancers that are considered prone to immune evasion. The molecular properties and phenotype of GBM make it an extraordinarily difficult malignancy to treat from an immunologic perspective. Its invasive nature behind the BBB could confer a relatively high degree of isolation from immune activity, allowing tumors to grow silently with limited immune surveillance. Unlike previous studies, we have sought to examine the efficacy of adoptive T-cell therapy using the D-270MG^FLuc/GFP^ xenograft, which we demonstrate possesses diffuse intraparenchymal and perivascular invasion, consistent with the histopathological hallmarks of human GBM. Furthermore, unlike cell lines engineered to express EGFRvIII, such as U87MG.ΔEGFR, D-270 MG was isolated from a primary tumor that naturally expressed EGFRvIII and has maintained expression *ex vivo* and *in vivo.* This lends greater credence to its more accurate recapitulation of the clinical scenario.

We demonstrate here that EGFRvIII^+^ CAR T cells recognize tumors naturally expressing EGFRvIII, such as D-270MG^FLuc/GFP^. We show that these EGFRvIII^+^ CAR T cells recognize tumor cells in an antigen-specific manner *in vitro* as measured by ICS (**Fig. 2c, d**). It is important to note that we measured a 10–15% frequency of IFN-γ^+^ cells in our *in vitro* ICS assays, which is less than our recorded frequency of CD3^+^ CAR^+^ T cells. This has been a consistent and expected result under the culture, transduction, and assay protocols described here. One explanation may be the varied differentiation states of CD3^+^ CAR^+^ T cells, since different stages of activation can alter the incubation time required for T-cell secretion of IFN-γ. We have found that longer incubation times (greater than the 18 h described here) and altered tumor : T cell ratios yield frequencies>50%, and we suspect that this is due to the inclusion of more cells occupying a greater spectrum of activation. However, we chose the assay conditions described here since incubation times>18 h decrease cell viability, and 18 h incubations have yielded consistent results to date [38].

Our data show that systemically delivered EGFRvIII^+^ CAR T cells have the capacity to migrate to invasive tumor deposits within the CNS. T-cell migration across endothelium requires molecular cues provided by chemokine-chemokine receptor interaction and engagement of adhesion molecules, which are thought to be independent of TCR engagement [39], [40]. The cross-reactivity of murine adhesion molecules and chemokines with human T cells and their chemokine receptors is known to be limited [41], and although this could have negatively impacted T-cell localization, we instead observed a substantial influx of T cells into the invasive tumor. We are currently evaluating the contribution of tumor-derived human chemokines and EGFRvIII-specificity on CAR T cells to further elucidate the steps required for effective T-cell migration across the BBB in the invasive areas of GBM.

Importantly, we show here that systemically administered EGFRvIII^+^ CAR T cells have the capacity to inhibit tumor growth and prolong survival of mice with established D-270MG^FLuc/GFP^ tumors (**Fig. 4a, b**). Despite the evidence of an effective, antigen-specific immune response, it is important to note that brain tumors ultimately continued to grow and caused death even in mice receiving EGFRvIII^+^ CAR T cells (**Fig. 4b**). Normalized BLI values from these treated mice eventually reached comparable values to untreated and CAR control groups, indicating tumor growth after a period of dormancy likely mediated by antitumor T-cell activity (**Fig. 4a**). One possible explanation is the loss or functional loss of EGFRvIII^+^ CAR T cells in this model. To test the antitumor efficacy of EGFRvIII^+^ CAR T cells *in vivo*, we chose an NSG mouse model, which has the advantage of evaluating promising preclinical therapies in an animal system using human tumor tissue. However, one major drawback of this approach is the fact that human T cells often have a limited lifespan and functional half-life in the murine background. For this reason, we sought to monitor T-cell persistence over time *in vivo* by BLI analysis. However, we unexpectedly found the administration of coelenterazine to be toxic at the manufacturer’s recommended dosage, and as such, terminated BLI measurements after day 9 (**Fig. 3a, b**). Our studies demonstrate that, despite this potential for CAR T cell loss, EGFRvIII^+^ CAR T cells were able to persist long enough to migrate to and mediate antitumor activity against invasive intracranial tumors. We are currently evaluating host conditioning regimens to support enhanced and long-term human T-cell survival and function in NSG mice, since these factors could, in theory, potentiate antitumor efficacy.

A second possible explanation for the eventual recurrence of tumor in our model is the concept of antigen-loss, wherein therapeutic pressure selects for tumor cells that do not express the target antigen. This explanation would be consistent with two recent clinical studies where recurrence in patients treated with CARs [42] or a vaccine targeting a single antigen [43] was characterized by outgrowth of antigen-loss variants. As such, given the theoretical limitations of targeting single tumor antigens, future efforts will likely focus on the identification of additional GBM-specific targets and multimodal therapies designed to target several antigens simultaneously through CAR-mediated or alternative T-cell-based approaches. Additional areas of further investigation may include determining factors involved in eliciting broader endogenous immune responses through mechanisms such as epitope spreading, which has emerged as a critical factor during clinical trials of immunotherapy for melanoma [44].

In the current study, we have demonstrated that systemically administered and tumor-specific EGFRvIII^+^ CAR T cells effectively migrate to areas of tumor invasion and mediate efficacy in a murine model of invasive human glioma. This work contributes to the rapidly growing literature supporting the utility of adoptively transferred CAR T cells targeting tumor-specific antigens as a potent treatment modality for invasive brain tumors.

## Materials and Methods

### Human GBM cell lines and xenografts

We utilized the previously described human glioma cell line, U87MG [29]–[31], which does not express EGFRvIII, and subline U87MG.ΔEGFR [33], [34], which was stably transfected to express EGFRvIII. We also utilized the D-270 MG cell line, which was propagated directly as a xenograft from a primary human GBM harvested from a patient and has previously been shown to naturally express EGFRvIII [20], [32]. Briefly, mechanically minced tumor tissue was enzymatically dissociated into single cells using the Papain Dissociation System. After wash, tissue was homogenized and passed through a 75 μm cell strainer, re-suspended with freezing medium containing 90% fetal bovine serum (FBS) and 10% dimethyl sulfoxide and frozen in individual vials using standard procedures. For further experimentation, cells were thawed at 37°C, washed, and counted with trypan blue per standard practice.

### Animals

NSG mice were obtained (Charles River Laboratories, Wilmington, MA) and bred under standard conditions at Duke University Medical Center (DUMC). Mice were kept and utilized under the accordance of protocols approved by the Duke University Institutional Animal Care and Use Committee (IACUC). All mice used in this study were healthy females between 6–8 weeks of age weighing 0.020–0.025 kg and were randomized to experimental or control groups. Mice were routinely monitored for health (every 2 days) and qualified for euthanasia if they demonstrated an inability to ambulate to food and water (i.e. in lateral recumbency and unable to right itself), or if they were unable to move forward two steps when prompted gently by touching a finger to the hind area. Moribund mice were humanely euthanized when they met these endpoints using CO_2_ asphyxiation followed by decapitation as approved by our Duke University IACUC protocol. In accordance with this protocol, mice did not receive any analgesics or anesthetics.

### Human PBLs

Human PBLs used in this study were obtained from normal volunteers at Duke University Medical Center. The use of PBLs was approved under protocol 0009043 by the Duke University School of Medicine Institutional Review Board (irb.mc.duke.edu). Approved protocols conform to the Declaration of Helsinki protocols. All patients signed a written informed consent. PBLs were cultured in AIM-V medium (Life Technologies, Grand Island, NY) supplemented with 10% human AB serum (Valley Biomedical Inc., Winchester, VA), 50 units/mL penicillin, 50 μg/mL streptomycin (Life Technologies, Grand Island, CA) and 300 IU/mL interleukin-2 (IL-2) and maintained at 37°C with 5% CO_2_.

### EGFRvIII^+^ CARs and extGLuc retroviral vector transduction

The extGLuc retroviral vectors were supplied by Renier Brentjens of Memorial Sloan-Kettering Cancer Center, New York, NY. The EGFRvIII^+^ CAR and extGLuc retroviral vectors were utilized to generate EGFRvIII^+^ CAR T cells and exGluc^+^ EGFRvIII^+^ CAR T cells. The transduction procedures have previously been described [36], [45], [46]. Briefly, peripheral blood mononuclear cells (PBMCs) from healthy donors and GBM patients (post-resection, prior to treatment) were thawed and cultured in AIM-V medium supplemented with 5% human AB serum, plus antibiotics, 300 IU/mL IL-2, and 50 ng/mL OKT-3. After 48 hours, T cells (0.25×10^6^/mL) were transduced with a retroviral supernatant containing either the extGLuc, EGFRvIII^+^ CAR, or both vectors spun onto RetroNectin (Takara Bio Inc, Japan) coated non-tissue culture treated 6-well plates twice on two consecutive days as described by the manufacturer. Transduced cells were allowed to expand in AIM-V medium as above, without OKT-3.

### Rapid Expansion Protocol

Transduced PBLs (or untransduced control PBLs from same donor) were expanded *in vitro* using rapid expansion protocol (REP) [47]. Briefly, T cells were cultured in complete AIM-V medium plus 10% human AB serum, 300 IU/mL IL-2, and 50 ng/mL OKT-3 in the presence of 100x excess 5000 rads irradiated allogeneic PBMC feeder cells, and allowed to expand 10–14 days.

### Lentiviral transduction of D-270 MG with firefly-luciferase-GFP gene

The D-270 MG tumor cell line was transduced with a lentiviral vector encoding the firefly luciferase (FLuc) and EGFP genes linked by 2A peptide driven by an internal murine stem cell virus (MSCV) promoter. Briefly, lentiviral vectors were generated by transient transfection of HEK 293T cells with a four-plasmid system [48]. Six hours post-transfection, plates were washed twice with phosphate-buffered saline (PBS) and 20 mL fresh medium was added. The supernatant was collected 30–48 hours post-transfection and cell debris was removed by centrifugation at 6000 *g* for 10 minutes, followed by filtration on 0.45 μm polyvinylidene fluoride filters. The lentiviral supernatant was kept at −80°C. D-270 MG cells were then quickly thawed at 37°C, washed, counted with trypan blue, and re-suspended in the lentiviral supernatant and zinc medium with 10% FBS and incubated at 37°C with 5% CO_2_ overnight. D-270MG^FLuc/GFP^ tumor cells were cell sorted based on GFP expression. Expression of FLuc was confirmed by data acquisition using the IVIS 100 *in vivo* BLI system (Caliper Life Sciences, Hopkinton, MA) coupled with Living Image software (PerkinElmer, Waltham, MA). EGFRvIII expression levels by D-270MG^FLuc/GFP^ and U87MG.ΔEGFR tumor cells were measured by qRT PCR as previously described [49].

### Cell surface CAR expression and ICS of transduced T cells

Cells were stained for EGFRvIII^+^ CARs to detect cell-surface expression using an EGFRvIII multimer-PE as previously described [50]. Negative staining controls were conducted by staining untransduced cells from the same donor. ICS was performed by co-culturing T cells with tumors 1∶1 over 18 hours with the BD GolgiPlug protein transport inhibitor containing brefeldin A (BD Sciences, San Jose, CA) in RPMI-1640 medium plus 10% FBS. Following co-culture, cells were submitted to surface staining for CD3, CD8 and intracellular IFN-γ stain using the BD Cytofix/Cytoperm method (BD Biosciences, San Jose, CA).

### Monitoring tumor growth and T-cell trafficking using BLI

To monitor tumor growth, 24 NSG mice underwent intracranial implantation of 1×10^4^ D-270MG^FLuc/GFP^ tumor cells in 5 μl PBS and methocell mixer using stereotactic coordinates 2 mm lateral and 4 mm intraparenchymal from the bregma on experimental day 0. Tumor growth was analyzed every three to five days for 26 days by BLI as previously described [51] until the study was terminated. Briefly, BLI was performed by injecting mice intraperitoneally with 150 mg D-luciferin/kg (Xenogen, Hopkinton, MA) 10 minutes prior to imaging and photon emission (photons s^−1^ cm^−2^ sr^−1^) was recorded. To monitor T-cell trafficking, T cells were transduced with the extGLuc or co-transduced with the EGFRvIII^+^ CAR and extGLuc retroviral vectors as described above (see *EGFRvIII^+^ CARs and extGLuc retroviral vector transduction*). Mice receiving T cells were injected with 5.0 - 10×10^6^ cells intravenously via the tail vein and imaged on days 2 and 9 after infusion. Briefly, 250 μg coelenterazine (NanoLight Technology, Pinetop, AZ) was injected IV in mice receiving either saline or EGFRvIII^+^ extGLuc^+^ CAR T cells and imaged within 90 seconds by measuring BLI. We obtained image data sets and measurement of signal intensity by using the IVIS 100 *in vivo* BLI system and through region of interest analysis using Living Image Software with normalized images displayed on each data set according to color intensity. Mean BLI values for control and treatment groups were calculated and plotted according to the corresponding day of imaging.

### H&E Staining and Immunohistochemistry

In order to evaluate tumor architecture and growth patterns, NSG mice received intracerebral tumor implants (D-270MG^FLuc/GFP^/1×10^4^ cells or U87MG.ΔEGFR/1×10^4^ cells) and were sacrificed after seven days. Brains were harvested, formalin-fixed and paraffin-embedded. 5 μm sections were stained with H&E. To evaluate T-cell migration to the invasive edge of tumor, we utilized two experimental groups, which included four tumor-bearing mice treated with saline or extGLuc^+^ EGFRvIII^+^ CAR 1×10^7^ T cells. Mice were euthanized on day 9. Brains were harvested, formalin-fixed and paraffin-embedded. 5 μm coronal sections were immunostained with rabbit anti-human CD3 antibody (Thermo Lab Vision, Clone RM9107S) as recommended by the manufacturer at a 1∶100 dilution and counterstained with hematoxylin.

### Statistical methods

Statistical differences in group percentage cytotoxicity and bioluminescence were evaluated by either a one-way analysis of variance (ANOVA) model or a two-way ANOVA model with interaction. The Kaplan Meier estimator was used to generate survival curves, and differences between survival curves were calculated using a generalized Wilcoxon test. Patterns of change in normalized BLI values on the log scale over time were evaluated using a mixed model that included main effects for time and treatment group along with a time interaction with treatment group. This model accounted for within animal correlation of measurements by using a 1^st^ degree autoregressive covariance structure.

## References

[1] Stupp R, Mason WP, van den Bent MJ, Weller M, Fisher B, et al (2005) Radiotherapy plus concomitant and adjuvant temozolomide for glioblastoma. N Engl J Med 352: 987–996. 15758009 10.1056/NEJMoa043330

[2] Imperato JP, Paleologos NA, Vick NA (1990) Effects of treatment on long-term survivors with malignant astrocytomas. Ann Neurol 28: 818–822. 2178330 10.1002/ana.410280614

[3] Johnson LA, Morgan RA, Dudley ME, Cassard L, Yang JC, et al (2009) Gene therapy with human and mouse T-cell receptors mediates cancer regression and targets normal tissues expressing cognate antigen. Blood 114: 535–546. 19451549 10.1182/blood-2009-03-211714 PMC2929689

[4] Hong JJ, Rosenberg SA, Dudley ME, Yang JC, White DE, et al (2010) Successful treatment of melanoma brain metastases with adoptive cell therapy. Clin Cancer Res 16: 4892–4898. 20719934 10.1158/1078-0432.CCR-10-1507 PMC6291850

[5] Robbins PF, Morgan RA, Feldman SA, Yang JC, Sherry RM, et al (2011) Tumor regression in patients with metastatic synovial cell sarcoma and melanoma using genetically engineered lymphocytes reactive with NY-ESO-1. J Clin Oncol 29: 917–924. 21282551 10.1200/JCO.2010.32.2537 PMC3068063

[6] Rosenberg SA (2011) Cell transfer immunotherapy for metastatic solid cancer—what clinicians need to know. Nat Rev Clin Oncol 8: 577–585. 21808266 10.1038/nrclinonc.2011.116 PMC6292196

[7] Kochenderfer JN, Dudley ME, Feldman SA, Wilson WH, Spaner DE, et al (2012) B-cell depletion and remissions of malignancy along with cytokine-associated toxicity in a clinical trial of anti-CD19 chimeric-antigen-receptor-transduced T cells. Blood 119: 2709–2720. 22160384 10.1182/blood-2011-10-384388 PMC3327450

[8] Rosenberg SA, Yang JC, Robbins PF, Wunderlich JR, Hwu P, et al (2003) Cell transfer therapy for cancer: lessons from sequential treatments of a patient with metastatic melanoma. J Immunother 26: 385–393. 12973027 10.1097/00002371-200309000-00001 PMC1764125

[9] Zitvogel L, Tesniere A, Kroemer G (2006) Cancer despite immunosurveillance: immunoselection and immunosubversion. Nat Rev Immunol 6: 715–727. 16977338 10.1038/nri1936

[10] Kalos M, June CH (2013) Adoptive T cell transfer for cancer immunotherapy in the era of synthetic biology. Immunity 39: 49–60. 23890063 10.1016/j.immuni.2013.07.002 PMC3809038

[11] Lamers CH, Sleijfer S, Vulto AG, Kruit WH, Kliffen M, et al (2006) Treatment of metastatic renal cell carcinoma with autologous T-lymphocytes genetically retargeted against carbonic anhydrase IX: first clinical experience. J Clin Oncol 24: e20–22. 16648493 10.1200/JCO.2006.05.9964

[12] Till BG, Jensen MC, Wang J, Qian X, Gopal AK, et al (2012) CD20-specific adoptive immunotherapy for lymphoma using a chimeric antigen receptor with both CD28 and 4-1BB domains: pilot clinical trial results. Blood 119: 3940–3950. 22308288 10.1182/blood-2011-10-387969 PMC3350361

[13] Pule MA, Savoldo B, Myers GD, Rossig C, Russell HV, et al (2008) Virus-specific T cells engineered to coexpress tumor-specific receptors: persistence and antitumor activity in individuals with neuroblastoma. Nat Med 14: 1264–1270. 18978797 10.1038/nm.1882 PMC2749734

[14] Brentjens RJ, Davila ML, Riviere I, Park J, Wang X, et al (2013) CD19-targeted T cells rapidly induce molecular remissions in adults with chemotherapy-refractory acute lymphoblastic leukemia. Sci Transl Med 5: 177ra138. 10.1126/scitranslmed.3005930 PMC3742551 23515080

[15] Porter DL, Levine BL, Kalos M, Bagg A, June CH (2011) Chimeric antigen receptor-modified T cells in chronic lymphoid leukemia. N Engl J Med 365: 725–733. 21830940 10.1056/NEJMoa1103849 PMC3387277

[16] Brentjens R, Yeh R, Bernal Y, Riviere I, Sadelain M (2010) Treatment of chronic lymphocytic leukemia with genetically targeted autologous T cells: case report of an unforeseen adverse event in a phase I clinical trial. Mol Ther 18: 666–668. 20357779 10.1038/mt.2010.31 PMC2862525

[17] Morgan RA, Yang JC, Kitano M, Dudley ME, Laurencot CM, et al (2010) Case report of a serious adverse event following the administration of T cells transduced with a chimeric antigen receptor recognizing ERBB2. Mol Ther 18: 843–851. 20179677 10.1038/mt.2010.24 PMC2862534

[18] Wikstrand CJ, Hale LP, Batra SK, Hill ML, Humphrey PA, et al (1995) Monoclonal antibodies against EGFRvIII are tumor specific and react with breast and lung carcinomas and malignant gliomas. Cancer Res 55: 3140–3148. 7606735

[19] Heimberger AB, Hlatky R, Suki D, Yang D, Weinberg J, et al (2005) Prognostic effect of epidermal growth factor receptor and EGFRvIII in glioblastoma multiforme patients. Clin Cancer Res 11: 1462–1466. 15746047 10.1158/1078-0432.CCR-04-1737

[20] Wong AJ, Ruppert JM, Bigner SH, Grzeschik CH, Humphrey PA, et al (1992) Structural alterations of the epidermal growth factor receptor gene in human gliomas. Proc Natl Acad Sci U S A 89: 2965–2969. 1557402 10.1073/pnas.89.7.2965 PMC48784

[21] Engelhardt B (2006) Molecular mechanisms involved in T cell migration across the blood-brain barrier. Journal of Neural Transmission 113: 477–485. 16550326 10.1007/s00702-005-0409-y

[22] Banks WA, Erickson MA (2010) The blood-brain barrier and immune function and dysfunction. Neurobiology of Disease 37: 26–32. 19664708 10.1016/j.nbd.2009.07.031

[23] Rascher G, Fischmann A, Kroger S, Duffner F, Grote EH, et al (2002) Extracellular matrix and the blood-brain barrier in glioblastoma multiforme: spatial segregation of tenascin and agrin. Acta Neuropathol 104: 85–91. 12070669 10.1007/s00401-002-0524-x

[24] Ajay D, Sanchez-Perez L, Choi BD, De Leon G, Sampson JH (2012) Immunotherapy with tumor vaccines for the treatment of malignant gliomas. Curr Drug Discov Technol 9: 237–255. 22339070 10.2174/157016312803305933

[25] Claes A, Idema AJ, Wesseling P (2007) Diffuse glioma growth: a guerilla war. Acta Neuropathol 114: 443–458. 17805551 10.1007/s00401-007-0293-7 PMC2039798

[26] Giese A, Westphal M (1996) Glioma invasion in the central nervous system. Neurosurgery 39: 235–250 discussion 250–232. 8832660 10.1097/00006123-199608000-00001

[27] Agarwal S, Manchanda P, Vogelbaum MA, Ohlfest JR, Elmquist WF (2013) Function of the blood-brain barrier and restriction of drug delivery to invasive glioma cells: findings in an orthotopic rat xenograft model of glioma. Drug Metab Dispos 41: 33–39. 23014761 10.1124/dmd.112.048322 PMC3533422

[28] Vauleon E, Avril T, Collet B, Mosser J, Quillien V, et al. (2010) Overview of Cellular Immunotherapy for Patients with Glioblastoma. Clinical and Developmental Immunology 2010. 10.1155/2010/689171 PMC2952949 20953324

[29] Hashizume R, Ozawa T, Dinca EB, Banerjee A, Prados MD, et al (2010) A human brainstem glioma xenograft model enabled for bioluminescence imaging. J Neurooncol 96: 151–159. 19585223 10.1007/s11060-009-9954-9 PMC2808534

[30] Miura FK, Alves MJ, Rocha MC, da Silva R, Oba-Shinjo SM, et al (2010) Xenograft transplantation of human malignant astrocytoma cells into immunodeficient rats: an experimental model of glioblastoma. Clinics (Sao Paulo) 65: 305–309. 20360922 10.1590/S1807-59322010000300011 PMC2845772

[31] Candolfi M, Curtin JF, Nichols WS, Muhammad AG, King GD, et al (2007) Intracranial glioblastoma models in preclinical neuro-oncology: neuropathological characterization and tumor progression. J Neurooncol 85: 133–148. 17874037 10.1007/s11060-007-9400-9 PMC2384236

[32] Bigner SH, Humphrey PA, Wong AJ, Vogelstein B, Mark J, et al (1990) Characterization of the epidermal growth factor receptor in human glioma cell lines and xenografts. Cancer Res 50: 8017–8022. 2253244

[33] Nishikawa R, Ji XD, Harmon RC, Lazar CS, Gill GN, et al (1994) A mutant epidermal growth factor receptor common in human glioma confers enhanced tumorigenicity. Proc Natl Acad Sci U S A 91: 7727–7731. 8052651 10.1073/pnas.91.16.7727 PMC44475

[34] Lal A, Glazer CA, Martinson HM, Friedman HS, Archer GE, et al (2002) Mutant epidermal growth factor receptor up-regulates molecular effectors of tumor invasion. Cancer Res 62: 3335–3339. 12067969

[35] Morgan RA, Johnson LA, Davis JL, Zheng Z, Woolard KD, et al (2012) Recognition of glioma stem cells by genetically modified T cells targeting EGFRvIII and development of adoptive cell therapy for glioma. Hum Gene Ther 23: 1043–1053. 22780919 10.1089/hum.2012.041 PMC3472555

[36] Santos EB, Yeh R, Lee J, Nikhamin Y, Punzalan B, et al (2009) Sensitive in vivo imaging of T cells using a membrane-bound Gaussia princeps luciferase. Nat Med 15: 338–344. 19219023 10.1038/nm.1930 PMC2837150

[37] Sadelain M, Brentjens R, Riviere I (2013) The basic principles of chimeric antigen receptor design. Cancer Discov 3: 388–398. 23550147 10.1158/2159-8290.CD-12-0548 PMC3667586

[38] Choi BD, Suryadevara CM, Gedeon PC, Herndon Ii JE, Sanchez-Perez L, et al (2014) Intracerebral delivery of a third generation EGFRvIII-specific chimeric antigen receptor is efficacious against human glioma. J Clin Neurosci 21: 189–190. 24054399 10.1016/j.jocn.2013.03.012 PMC3867597

[39] Engelhardt B, Ransohoff RM (2012) Capture, crawl, cross: the T cell code to breach the blood-brain barriers. Trends Immunol 33: 579–589. 22926201 10.1016/j.it.2012.07.004

[40] Ransohoff RM (2009) Chemokines and chemokine receptors: standing at the crossroads of immunobiology and neurobiology. Immunity 31: 711–721. 19836265 10.1016/j.immuni.2009.09.010 PMC2787682

[41] Mestas J, Hughes CC (2004) Of mice and not men: differences between mouse and human immunology. J Immunol 172: 2731–2738. 14978070 10.4049/jimmunol.172.5.2731

[42] Grupp SA, Kalos M, Barrett D, Aplenc R, Porter DL, et al (2013) Chimeric antigen receptor-modified T cells for acute lymphoid leukemia. N Engl J Med 368: 1509–1518. 23527958 10.1056/NEJMoa1215134 PMC4058440

[43] Sampson JH, Heimberger AB, Archer GE, Aldape KD, Friedman AH, et al (2010) Immunologic escape after prolonged progression-free survival with epidermal growth factor receptor variant III peptide vaccination in patients with newly diagnosed glioblastoma. J Clin Oncol 28: 4722–4729. 20921459 10.1200/JCO.2010.28.6963 PMC3020702

[44] Butterfield LH, Ribas A, Dissette VB, Amarnani SN, Vu HT, et al (2003) Determinant spreading associated with clinical response in dendritic cell-based immunotherapy for malignant melanoma. Clin Cancer Res 9: 998–1008. 12631598

[45] Hughes MS, Yu YY, Dudley ME, Zheng Z, Robbins PF, et al (2005) Transfer of a TCR gene derived from a patient with a marked antitumor response conveys highly active T-cell effector functions. Hum Gene Ther 16: 457–472. 15871677 10.1089/hum.2005.16.457 PMC1476695

[46] Morgan RA, Dudley ME, Yu YY, Zheng Z, Robbins PF, et al (2003) High efficiency TCR gene transfer into primary human lymphocytes affords avid recognition of melanoma tumor antigen glycoprotein 100 and does not alter the recognition of autologous melanoma antigens. J Immunol 171: 3287–3295. 12960359 10.4049/jimmunol.171.6.3287 PMC2248799

[47] Riddell SR, Greenberg PD (1990) The use of anti-CD3 and anti-CD28 monoclonal antibodies to clone and expand human antigen-specific T cells. J Immunol Methods 128: 189–201. 1691237 10.1016/0022-1759(90)90210-m

[48] Yang S, Cohen CJ, Peng PD, Zhao Y, Cassard L, et al (2008) Development of optimal bicistronic lentiviral vectors facilitates high-level TCR gene expression and robust tumor cell recognition. Gene Ther 15: 1411–1423. 18496571 10.1038/gt.2008.90 PMC2684456

[49] Yoshimoto K, Dang J, Zhu S, Nathanson D, Huang T, et al (2008) Development of a real-time RT-PCR assay for detecting EGFRvIII in glioblastoma samples. Clin Cancer Res 14: 488–493. 18223223 10.1158/1078-0432.CCR-07-1966

[50] Sampson JH, Choi BD, Sanchez-Perez L, Suryadevara CM, Snyder DJ, et al. (2013) EGFRvIII mCAR-modified T cell therapy cures mice with established intracerebral glioma and generates host immunity against tumor-antigen loss. Clin Cancer Res. 10.1158/1078-0432.CCR-13-0709 PMC3943170 24352643

[51] Szentirmai O, Baker CH, Lin N, Szucs S, Takahashi M, et al (2006) Noninvasive bioluminescence imaging of luciferase expressing intracranial U87 xenografts: correlation with magnetic resonance imaging determined tumor volume and longitudinal use in assessing tumor growth and antiangiogenic treatment effect. Neurosurgery 58: 365–372 discussion 365–372. 16462491 10.1227/01.NEU.0000195114.24819.4F

